# The moderating effect of prefrontal response to sleep-related stimuli on the association between depression and sleep disturbance in insomnia disorder

**DOI:** 10.1038/s41598-022-22652-9

**Published:** 2022-10-22

**Authors:** Mi Hyun Lee, Kyung Hwa Lee, Seong Min Oh, Min Cheol Seo, Hayoung Lee, Jeong Eun Jeon, Yu Jin Lee

**Affiliations:** 1 Department of Psychiatry and Center for Sleep and Chronobiology, Seoul National University College of Medicine, Seoul, Republic of Korea; 2 Division of Child and Adolescent Psychiatry, Department of Psychiatry, Seoul National University Hospital, Seoul, Republic of Korea; 3 Department of Psychiatry, Seoul Topclass Psychiatry Clinic, Seoul, Republic of Korea

**Keywords:** Depression, Sleep, Depression

## Abstract

We investigated differences in brain activity in response to sleep-related pictures between chronic insomnia disorder (CID) patients and good sleepers (GS), and examined whether brain activity moderated the relationship between depressive symptoms and sleep disturbance in CID patients and GS. This study included 43 patients diagnosed with CID, based on the International Classification of Sleep Disorders-3, and 42 GS. The participants kept a sleep diary, underwent nocturnal polysomnography to measure sleep parameters, and completed self-report questionnaires to assess sleep and psychiatric symptoms. They underwent functional magnetic resonance imaging (fMRI) to examine differences in brain activity in response to sleep-related pictures compared to neutral pictures. A moderated moderation analysis was performed to investigate the moderating role of brain responses to sleep-related pictures in the association between depressive symptoms and sleep disturbance. Compared to GS, the brain responses to sleep-related stimuli were significantly lower in CID patients in the right lateral prefrontal cortex (LPFC) and dorsomedial prefrontal cortex (DMPFC). More severe depressive symptoms were significantly associated with longer sleep latency only when LPFC activity was low in CID patients, but not in GS. LPFC hypoactivity in response to sleep-related stimuli in CID patients could moderate the relationship between depression and sleep disturbance.

## Introduction

Insomnia is a common symptom and important risk factor for depression^1^. The relationship between insomnia and depression is bidirectional, and they have both been linked to structural and functional changes in the frontal cortex^2,3^. A large cohort study of 1,053 major depressive disorder (MDD) patients demonstrated that patients with more severe insomnia had smaller cortical surface areas in several frontal regions^4^. A recent neuroimaging review reported similar prefrontal abnormalities in MDD and insomnia^5^. However, structural and functional impairments in depressive disorder and insomnia disorder were inconsistent in two different neuroimaging meta-analyses^6,7^.

Espie et al.^8^ demonstrated that attentional bias toward external stimuli contributed to chronic insomnia through the attention-intention-effort model (AIE). Sleep-related attentional bias is defined as the propensity to selectively focus on sleep-related stimuli rather than neutral information. Selective attention to sleep-related stimuli inhibits sleep automaticity in individuals with hyperarousal insomnia, which leads to chronic insomnia due to sleep-related maladaptive behavior^9^. Hyperarousal, or overactivation of the arousal system, is a feature of both insomnia and depression^10,11^. Liu et al.^12^ reported an increase in the resting state amplitude of low-frequency fluctuation (ALFF) in the inferior frontal gyrus in relation to hyperarousal insomnia in MDD patients. Impairment of the salience network in depressive disorders has been described previously^13,14^. The amygdala, an important structure of the salience network, becomes hyper-responsive to negative stimuli in depressive patients, which leads to emotional dysregulation^4,15^. Therefore, it is necessary to explore the shared brain networks involved in the hyperarousal system and attentional bias, and their roles in the pathogenesis of insomnia and depression.

A few studies on sleep-related stimuli in insomnia patients have used functional magnetic resonance imaging (fMRI) to explore attentional bias. Moreover, the results have been inconsistent and none of these studies included depressive patients. Patients with psychophysiological insomnia exhibited greater activation of the precentral and prefrontal cortices in response to sleep-related pictures compared to good sleepers (GS) and the increased responses were reduced after cognitive-behavioral therapy^16^. This study supported a hyperarousal model of insomnia. Another study of 14 insomnia patients demonstrated decreased regional activity in the left middle temporal and left middle occipital gyri in response to sleep-related sounds following cognitive behavioral treatment for insomnia^17^. Spiegelhalder et al.^18^ suggested the use of sleep-related pictorial stimuli because the brain activity in insomnia patients, assessed using the emotional Stroop test, did not differ from that in controls. Brain imaging studies of patients with insomnia have examined dysfunction of the limbic system as well as the prefrontal region. Baglioni et al.^19^ demonstrated increased activity of the amygdala in response to insomnia-related images in insomnia patients. Huang et al.^20^ reported decreased functional connectivity between the amygdala and insula, striatum, and thalamus in insomnia patients in comparison to controls in the resting state. A resting state fMRI study of healthy adults with insomnia showed that sleep disturbance was associated with altered activity in the thalamus of the hyperarousal network^21^.

Previous fMRI studies using sleep-related stimuli had several limitations. None of the studies investigated the role of attentional bias, which is related to the hyperarousal system, in the relationship between depressive and insomnia symptoms. In addition, the number of patients was small and other sleep disorders, including obstructive sleep apnea and periodic limb movement disorder, were not excluded through polysomnography (PSG).

The present study aimed to investigate differences in brain activity in response to sleep-related pictures between patients with chronic insomnia disorder (CID) and GS, and to determine whether brain activity in specific regions moderated the relationship between depression and sleep disturbance. We tested the following two hypotheses. First, CID patients would show different activation in response to sleep-related pictures in insomnia-related regions compared to GS, especially in the frontal cortex and limbic structures. Second, we hypothesized that insomnia would be more severe in the presence of severe depressive symptoms and abnormal brain function.

## Results

### Demographic, clinical, and sleep variables

Table 1 summarizes the clinical and sleep variables of all participants. There were no significant differences in age (*P* = 0.067) or sex (*P* = 0.208) between CID patients and GS. However, CID patients had higher Pittsburgh Sleep Quality Index (PSQI), Beck Depression Inventory (BDI), Beck Anxiety Inventory (BAI), and Dysfunctional Beliefs and Attitudes about Sleep Scale-16 (DBAS-16) scores compared to GS (*P* < 0.05 for all). The range of BDI scores was 1–25 in the CID patients and 0–13 in GS. The median BDI value was 6 in both groups. The nocturnal PSG results showed that CID patients had a lower total sleep time (*P* = 0.014) and sleep efficiency (*P* = 0.007), and a longer wake time after sleep onset (WASO) (*P* = 0.011) compared to GS. The total sleep time and sleep efficiency, derived from the sleep diary, were significantly lower in CID patients than GS. The sleep latency was significantly higher (*P* < 0.001) and the WASO was longer in CID patients compared to GS, although these differences were not statistically significant.

**Table 1:** Comparison of demographics, scores on self-report questionnaires, and polysomnography and sleep diary sleep variables between the insomnia patients and good sleepers.

	Chronic insomnia disorder (n = 43) (M ± SD)	Good sleepers (n = 42) (M ± SD)	t or chi-squared values	*P*-value
**Demographics**				
Age (years)	44.6 ± 13.9	39.3 ± 11.9	1.86	0.067
Females, n (%)	33 (76.7%)	27 (64.3%)	1.59	0.208
**Self-report questionnaire**				
ESS	6.8 ± 4.5	6.5 ± 3.8	0.24	0.809
PSQI	12.6 ± 4.1	4.2 ± 1.8	12.31	**< 0.001**
BDI	10.8 ± 9.8	4.2 ± 5.1	3.93	**< 0.001**
BAI	10.4 ± 10.5	3.0 ± 3.7	4.35	**< 0.001**
DBAS	131.2 ± 58.2	92.6 ± 56.4	3.09	**0.003**
**Polysomnography**				
TIB (min)	438.1 ± 66.2	460.4 ± 34.9	− 1.91	0.061
TST (min)	386.8 ± 77.1	422.8 ± 49.8	− 2.51	**0.014**
SL (min)	17.1 ± 21.5	11.2 ± 10.2	1.58	0.119
SE (%)	83.6 ± 12.2	89.8 ± 7.3	− 2.79	**0.007**
WASO (min)	54.7 ± 37.9	35.9 ± 26.0	2.62	**0.011**
REML (min)	98.8 ± 59.1	113.1 ± 53.9	− 1.15	0.256
N1 (%)	13.8 ± 6.3	12.0 ± 5.3	1.40	0.172
N2 (%)	56.5 ± 9.2	58.0 ± 10.7	− 0.68	0.498
N3 (%)	8.8 ± 8.2	8.4 ± 6.7	0.25	0.801
REM (%)	24.5 ± 23.7	21.6 ± 5.7	0.79	0.436
AHI (/h)	2.4 ± 2.7	3.2 ± 4.0	− 1.03	0.308
PLMI (/h)	4.2 ± 6.0	2.6 ± 3.8	1.09	0.286
**Sleep diary**^a^				
TIB (min)	473.5 ± 304.7	470.7 ± 62.9	0.06	0.953
TST (min)	305.1 ± 91.4	419.5 ± 48.1	− 7.14	**< 0.001**
SL (min)	51.6 ± 60.6	13.0 ± 13.3	4.07	**< 0.001**
SE (%)	70.1 ± 18.3	90.1 ± 7.2	− 6.58	**< 0.001**
WASO (min)	114.5 ± 311.6	18.7 ± 16.6	2.01	0.051

*SD* standard deviation, *ESS* epworth sleepiness scale, *PSQI* Pittsburgh sleep quality index, *BDI* Beck depression inventory, *BAI* Beck anxiety inventory, *DBAS* brief version of the Dysfunctional Beliefs and Attitudes about Sleep Scale-16, *TIB* time in bed, *TST* total sleep time, *SL* sleep latency, *SE* sleep efficiency, *WASO* wake after sleep onset, *REML* REM latency, *AHI* apnea–hypopnea index, *PLMI* periodic limb movement index.

^a^Sleep diary data were available for 43 CID patients and 37 GS. Significant values are in bold.

### Brain activation in response to sleep-related pictures

**Figure 1:**
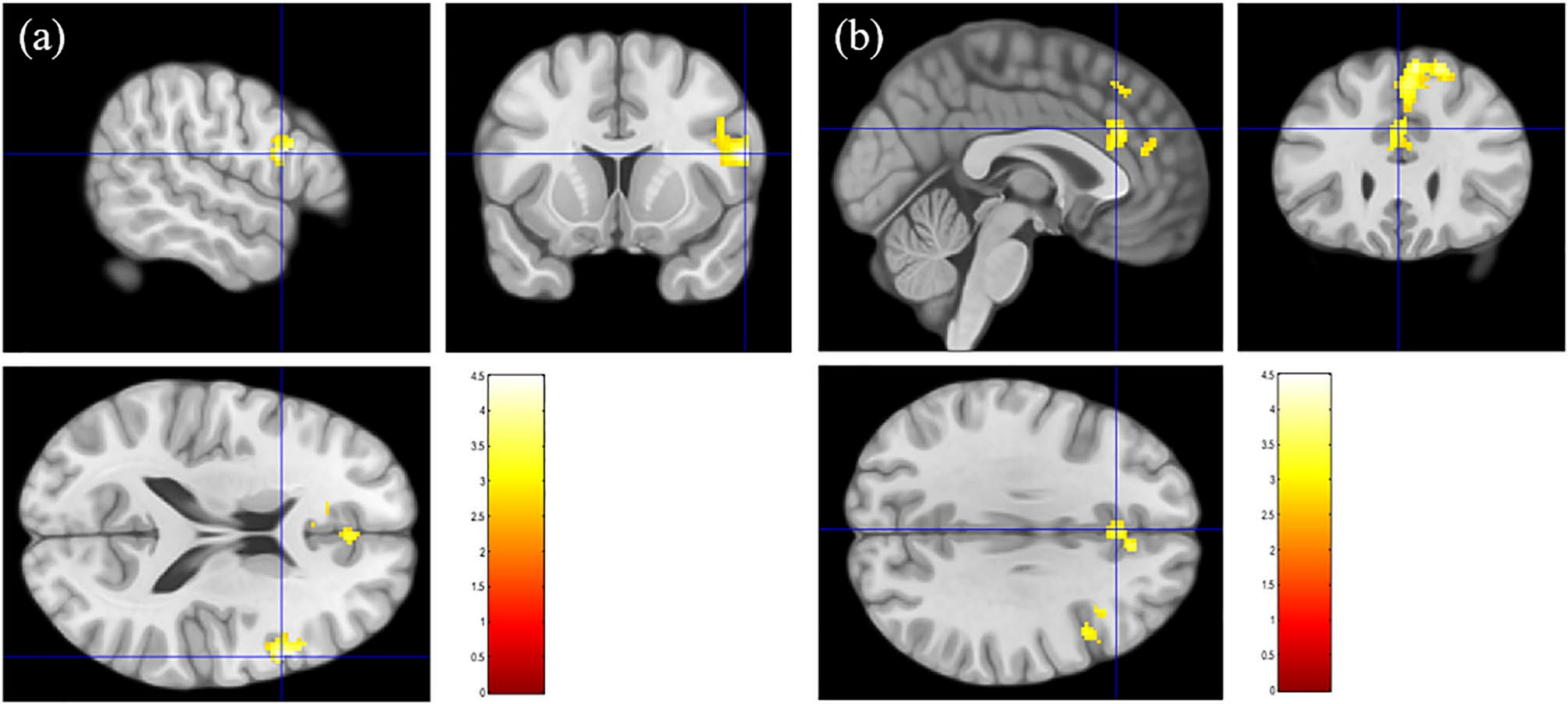
Brain areas showing less activation in response to sleep-related pictures in the insomnia group. Brain regions that showed more activation in good sleepers than insomnia patients in response to sleep-related pictures. Higher levels of brain activation (red and yellow) were observed in the right inferior frontal (**a**) and superior medial frontal (**b**) gyri in the good sleepers (FDR correction; *p* < 0.05).

Brain activation in response to sleep-related pictures was significantly lower in CID patients than in GS in prefrontal regions (Table 2, Fig. 1) (false discovery rate [FDR] corrected *P* < 0.05), including the right lateral prefrontal cortex (LPFC), dorsomedial prefrontal cortex (DMPFC), and anterior cingulate cortex (ACC). There were no regions with significantly greater activation in CID patients compared to GS in response to sleep-related pictures. CID showed shorter sleep duration (i.e., total sleep time measured by PSG and sleep diary) than GS (see Table 1). Analyses of covariance were conducted to examine whether sleep duration (i.e., total sleep time) measured by PSG and sleep diary affected group differences in prefrontal response to sleep-related pictures. After controlling for sleep duration, most findings of the group differences remained significant (*P* < 0.01). However, the group difference in DMPFC response to sleep pictures was marginally significant (*P* = 0.07) after controlling for total sleep time measured by sleep diary.

**Table 2:** Comparison of brain activation in response to sleep-related pictures between the insomnia patients and good sleepers.

Cluster	Region		Number of voxels in the region	Cluster size	MNI coordinates			Peak
		H		(voxels)	x	y	z	T
**Insomnia > good sleeper in the “sleep-related pictures”– “neutral pictures” contrast**								
No significant regions identified								
**Insomnia < good sleeper in the in the “sleep-related pictures”– “neutral pictures” contrast**								
1	Inferior frontal gyrus	R	207	362	56	12	18	4.49
	Middle frontal gyrus	R	106					
2	Superior medial frontal gyrus	R	138	620	6	44	24	4.01
	Anterior cingulum	L	121					
	Superior medial frontal gyrus	L	112					
	Superior frontal gyrus	R	94					
	Anterior cingulum	R	66

### Moderation analysis

Parameter estimates were extracted from clusters (right LPFC, DMPFC, and ACC) that showed significant differences between groups.

**Figure 2:**
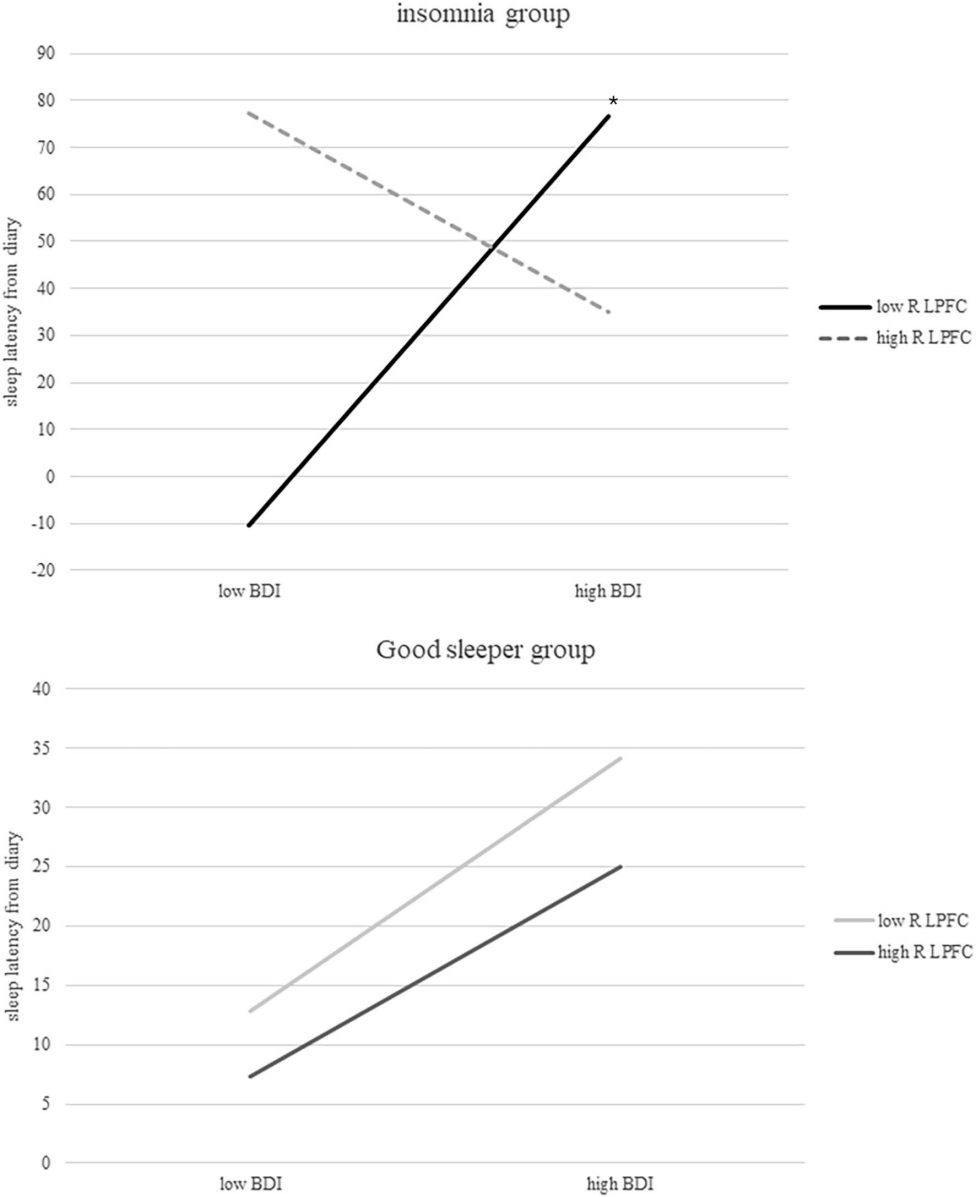
Interaction between BDI score and sleep latency (based on sleep diaries) according to the degree of right inferior frontal activation. BDI, Beck Depression Inventory; R LPFC, right lateral prefrontal cortex. * *P* < 0.0001.

Group (CID vs. GS) moderated the interaction between BDI score and right LPFC activity as predictors of sleep disturbance (Table 3). The moderating role of brain activity response to sleep-related pictures in the relationship between depression and sleep disturbance was significant in CID patients but not in GS. There was a significant, positive correlation between BDI and sleep latency determined from the sleep diary in CID patients with low-level right LPFC activity, but not in CID patients with high-level right LPFC activity (Fig. 2). No such significant correlations were found in GS. An analysis of DMPFC and ACC activity as moderators yielded no significant results.

**Table 3:** Correlation coefficients and 95% confidence intervals obtained via moderated moderation analysis of factors predicting sleep latency based on sleep diaries.

Model summary	R	R^2^	MSE	F	df	*P*
	0.6726	0.4524	1466.8	7.3314	8.71	**< 0.0001**

Model summary	Coeff	se	t	LLCI	ULCI	*P*
1. BDI	1.3097	0.8117	1.6135	− 0.3088	2.9281	0.1111
2.BDI × Right inferior frontal activation	− 7.4782	3.2460	− 2.3038	− 13.9506	− 1.0057	0.0242
3.BDI × Right inferior frontal activation × group	13.2115	6.5285	2.0237	0.1940	26.2290	0.0468

	Level of brain activation	Effect	se	t	*P*	
**Conditional effects of depression on insomnia symptom according to brain activation and group**						
Chronic insomnia group	Low (− 1SD)	5.4175	0.9405	5.7600	**< 0.0001**	
	High (+ 1SD)	− 2.6221	1.9337	− 1.3560	0.1794	
Good sleeper group	Low	1.3189	2.2784	0.5789	0.5645	
	High	1.0959	1.8294	0.5990	0.5511	

Model 1: main effects of BDI.

Model 2: interaction of BDI and activity of right inferior frontal region.

Model 3: three-way interaction of BDI, activity of right inferior frontal region, and group (CID patients and GS).

*LLCI* lower level confidence interval, *ULCI* upper level confidence interval, *BDI* beck depression inventory.

Significant values are in bold.

### Additional analyses

#### ROI-based analysis for group differences in the amygdala and anterior insula

The whole brain t test did not show any between-group differences in limbic structure activity, including the amygdala and anterior insula, in response to sleep-related pictures. Given that the amygdala and anterior insula are relatively small regions, FDR correction for multiple comparisons could hinder the identification of activity in these regions. Therefore, we performed a region of interest (ROI)-based analysis using a priori-defined regions of the amygdala and anterior insula. The ROIs were defined using automated anatomical labeling. Mean parameter estimates from each ROI were extracted and tested for between-group differences using a two-sample t test. The results showed no significant between-group differences in amygdala or anterior insula responses to sleep-related pictures vs. neutral pictures between CID patients and GS.

#### Moderation analysis using PSG data

Additional moderation analyses using PSG data were conducted to examine whether there were significant moderation effects of brain activity between BDI scores and sleep parameters collected from PSG in each CID patients and GS. The analyses did not find significant moderation effects.

## Discussion

In the present study, brain activation in response to sleep-related pictures was significantly lower in prefrontal areas, including the right LPFC, DMPFC, and ACC, in CID patients than in GS. There was a positive association between depressive symptoms and sleep latency determined from the sleep diary only when right LPFC activity was low in CID patients. The association between depressive symptoms and sleep latency was not observed in CID patients with high LPFC activity or in GS regardless of brain activity. Right LPFC hypoactivity in response to sleep-related stimuli moderated the relationship between depressive symptoms and sleep disturbance in CID patients.

Previous studies demonstrated decreased prefrontal cortex (PFC) activity in insomnia patients during cognitive tasks. Insomnia patients showed hypoactivation of medial and inferior prefrontal cortical areas compared to controls during the performance of category and letter fluency tasks^22^. Drummond et al.^23^ reported lower right dorsolateral PFC activation in insomnia patients with increasing task difficulty. Prefrontal regions contribute to executive functions, including attention, problem solving, and planning^24^. Reduced PFC activation in insomnia patients has been associated with cognitive disinhibition, such as rumination and intrusive thoughts^25^. Our study used sleep-related pictures, which are thought to have relatively low cognitive demand, but CID patients still showed low PFC activation. The low PFC activation in response to sleep-related stimuli in CID patients represents abnormal brain function and can be considered a neurobiological marker for insomnia.

It is possible that lower PFC activation in CID patients may be related to sleep loss, which often reported in CID patients. Previous studies have shown that sleep deprivation itself (sleep loss) is associated with frontal hypoactivation during performing tasks. A meta-analysis of 11 sleep deprivation neuroimaging showed that the activity of the frontoparietal attention network including the prefrontal cortex and intraparietal sulcus significantly decreased during different attention tasks after sleep deprivation^26^. Sleep deprivation was also related to reduced activity of the frontoparietal network and the intrusion of the activity in the default mode network during working memory task^27^. Given that our CID patients showed short sleep duration, we conducted additional analyses to examine the effect of sleep loss in prefrontal differences between CID and GS. As we reported above, most findings remained significant or marginally significant after controlling for sleep loss.

Insomnia patients often complain of depressive symptoms, and insomnia and depression are known to have a bidirectional relationship^1^. These neurobiological markers in CID patients may moderate the relationship between depressive symptoms and insomnia. The results of moderated moderation analyses showed that low LPFC activity played a regulatory role in the association between depressive symptoms and sleep disturbance in CID patients in the present study. Our findings suggest that severe depressive symptoms and lower PFC activity are associated with longer subjective sleep latency. Previous studies have demonstrated that reduced cognitive function is characteristic of patients with depressive disorders, which is reflected in LPFC hypoactivation^28^. When depressive symptoms are severe, cognitive function declines and attentional bias may occur in relation to symptoms of insomnia. Our findings highlight the importance of LPFC function in the relationship between depressive symptoms and sleep disturbance in CID patients, suggesting that improvements in PFC function and the alleviation of depression may be important in the treatment of insomnia.

Neuroimaging studies have demonstrated PFC impairment in top-down modulation of emotional processing when sleep is disrupted^29^. When viewing negative emotional stimuli, a sleep deprivation group exhibited greater activation of the amygdala compared to the control group, while increased limbic activity was associated with decreased functional connectivity with the medial PFC^30^. Motomura et al.^31^ reported that sleep debt reduced functional connectivity between the amygdala and ventromedial PFC in an fMRI study using fearful face stimuli. Furthermore, Banks et al.^32^ reported that coupling of the medial PFC and amygdala moderated emotion regulation in reappraisal tasks. An analysis of appraisal processes using fMRI revealed that the right ventrolateral PFC was associated with emotion generation and regulation^33^. The right LPFC, which is a key node in the salience network, significantly influenced the insomnia symptoms associated with MDD^12^.

Hyperarousal is the main pathophysiology of insomnia, although no significant hyperactivation of key elements of the salience network, such as the amygdala and anterior insula, was found in our insomnia group. Additional ROI analysis of amygdala and anterior insula activity showed no significant differences between the CID patients and GS, which would have been because the degree of activation in these regions was very small in response to sleep-related pictures. In this study, activation in response to sleep-related pictures was significantly lower in CID patients than GS in the right LPFC, DMPFC, and ACC. In contrast, a previous fMRI study demonstrated higher activation of the prefrontal and precentral regions in response to sleep-related pictures in 14 psychophysiological insomnia patients compared to GS^16^. These discrepancies were probably due to differences in participant characteristics. Kim et al.^16^ reported no significant differences in BDI scores between insomnia and GS groups, whereas the difference in our study was significant. Insomnia patients with more severe depressive symptoms, demonstrated by higher BDI scores, were enrolled in this study. In addition, the study population was larger and we used a diagnosis of chronic insomnia based on the International Classification of Sleep Disorders-Third Edition (ICSD-3) rather than psychophysiological insomnia based on the ICSD-2.

The anxiety accompanying insomnia could be a considerable clinical factor related to rumination and worry^34^. We found a significant difference in BAI scores between CID patients and GS. However, moderation analysis of BAI scores as an independent variable showed that prefrontal brain activity did not significantly moderate the relationship between anxiety and sleep disturbance in either group. This result was not consistent with moderation analysis results using BDI scores. One possible reason is that depression may be more manifest than anxiety in insomnia patients. In support of this possibility, in several epidemiological studies, insomnia disorder was more strongly associated with depressive disorder than with anxiety disorder. For example, insomnia had stronger predictive power in relation to future depression (OR = 3.51) compared to future anxiety in a general population prospective study^35^. In addition, an epidemiological study of adolescents showed that prior insomnia was associated with the onset of depressive disorder, but no significant association was observed with the onset of anxiety disorder^36^.

Meanwhile, when further analysis was performed using PSG data, which is the objective sleep parameter, a significant moderation effect of brain activity between sleep disturbance and depression was not observed. It is because one-night polysomnography data may be insufficient to reflect usual sleep disturbance in insomnia patients^37^. Furthermore, these finding could be due to the first night effect of the participants characterized by decreased total sleep time and lower sleep efficiencies on the first night of PSG^38^.

The present study has several limitations. First, the severity of depressive symptoms was evaluated subjectively, and detailed evaluations of the duration, recurrence, and age at onset of depression were insufficient. A comprehensive mood evaluation should be performed in future studies. Second, although non-emotional sleep-related images were used, they could still cause distress to insomnia patients. Emotional and arousal levels should be controlled for more accurate assessment of between-group differences in future studies. Third, we excluded patients with MDD and negative results may be observed in further analyses performed in the limbic region. Our study primarily focused on the neural characteristics of attentional bias to sleep-related stimuli in CID patients. Including subjects with both CID and MDD may be difficult with regard to controlling for the effects of depressive symptoms on neural responses to sleep-related stimuli. Despite the relatively low range (mostly mild levels) of depressive symptoms in CID, we examined how the neural response to sleep-related stimuli moderated the relationship between depressive symptoms and sleep disturbance in patients with chronic insomnia. In addition, to overcome the validity issue that arises because the BDI score contains an insomnia item, we also analyzed the BDI score with exclusion of the 16th sub-item related to insomnia, and the results showed the same significant moderation effect.

This is the first fMRI study to explore the roles of possible mediating factors in the relationship between sleep disturbance and depressive symptoms. Our findings suggest that prefrontal hypoactivity in insomnia patients moderates the association between depressive symptoms and sleep disturbance in the presence of sleep-related stimuli. These results could improve our understanding of the shared neurobiological mechanisms underlying depression and insomnia.

## Methods

### Participants

Initially, 46 CID patients and 45 GS (27 women; mean age = 39.3 ± 11.9 years) were enrolled. The period of recruitment and data collection were from May 11, 2014, to July 14, 2020. Patients aged 18–65 years who met ICSD-3 criteria for CID were recruited from an outpatient clinic at the Department of Psychiatry and from the Center for Sleep and Chronobiology at Seoul National University Hospital^39^. Moreover, 23 GS were recruited from the advertisement. The exclusion criteria were as follows: history of serious neurological or medical illness; current neurological or medical illness; other major psychiatric disorders, including cognitive impairments and personality disorders, as defined by the Diagnostic and Statistical Manual of Mental Disorders-Fourth Edition (DSM-IV); sleep disorders other than CID, such as obstructive sleep apnea (i.e., apnea–hypopnea index [AHI] ≥ 15) and periodic limb movement disorder (i.e., periodic limb movement index ≥ 15), based on ICSD-3 criteria; shiftwork; contraindications to MRI; and pregnancy. The study protocol was approved by the Ethics Committee of Seoul National University Hospital, in accordance with the Declaration of Helsinki. A complete description of the study protocol was provided to all participants and written informed consent was obtained.

The participants were screened for psychiatric disorders by trained psychologists using the Structural and Clinical Interviews for DSM-IV axis I disorders^40^. Therefore, participants who met the diagnostic criteria for MDD through a clinical interview were excluded. Participants were instructed to stop taking any medications that could influence sleep or circadian rhythms, such as sedatives, hypnotics, and antidepressants. Individuals on these medications could participate in the study after a drug washout period of at least 5 days. Among the 46 CID patients, 3 were excluded due to periodic limb movement disorders on nocturnal PSG, while 3 of the 45 GS were excluded due to obstructive sleep apnea (*n* = 2) or periodic limb movement disorder (*n* = 1). Finally, 43 CID patients (33 women; mean age = 44.6 ± 13.9 years) and 42 GS (27 women; mean age = 39.3 ± 11.9 years) were included in the analysis. Two patients in the CID group and none in the GS group were taking zolpidem at the time of recruitment. Participants taking zolpidem discontinued before the study after consulting with a psychiatrist. None of the subjects were taking antidepressants, sedatives, or hypnotics.

### Nocturnal polysomnography

At the Center for Sleep and Chronobiology of Seoul National University Hospital, overnight PSG (Profusion3; Compumedics, Charlotte, NC, USA) data, which consisted of EEG (with electrodes at F3, F4, C3, C4, O1, and O2, using A1 and A2 as reference sites), bilateral electrooculography (EOG), single-lead electrocardiography (ECG), submentalis and bilateral anterior tibialis electromyography (EMG), airflow measurements via nasal pressure transducers and oronasal thermal sensors, respiratory inductance plethysmography to monitor chest and abdominal movements, and finger pulse oximetry, were analyzed. PSG was scored by experienced technicians and physicians in accordance with the American Academy of Sleep Medicine (AASM) recommendations. The AHI, periodic limb movement index, time in bed, total sleep time, sleep latency, sleep efficiency, WASO, REM latency, and sleep percentage were recorded in each stage.

### Clinical assessment

All participants completed self-report questionnaires, including the Epworth Sleepiness Scale (ESS), PSQI, BDI, BAI, and DBAS-16. The ESS, which is widely used to measure sleep propensity, was applied to assess daytime sleepiness^41^. The PSQI is a self-report questionnaire that measures overall quality of sleep^42^. The BDI and BAI were used to measure the severity of depressive symptoms and anxiety, respectively^43,44^. The DBAS-16 is a self-report questionnaire on sleep-disruptive cognition, such as faulty appraisals, unrealistic expectations, and perceptual bias regarding sleep^45^. All participants were instructed to keep a 1-week sleep diary, which included time in bed, total sleep time, sleep latency, sleep efficiency, and WASO. The average of the 1-week sleep diary written by the subject every day was used for the analysis.

#### fMRI experimental procedure

The fMRI experiment had a block design, and consisted of randomly intermixed sleep and neutral blocks. We selected 28 sleep-related and 28 neutral pictures from the Internet as stimuli. The pictures were presented to 25 insomnia patients for validation, and classified as “sleep-related” if they were identified as such by more than 80% of the patients. The insomnia patients who participated in the validation study, which was conducted separately, were not included in this study. Both the sleep-related and neutral pictures were designed to not trigger or depict any specific emotions. There were no emotional or facial expressions in the sleep-related and neutral pictures, and they were matched for size and brightness. This validation process was described previously by Kim et al.^16^ Each block began with fixation for 1 s followed by a 12-s picture presentation, 4-s dot sign, and 2-s sleep rating. In each block, four sleep-related or neutral pictures were presented for 3 s each. After the picture presentation phase (12 s in total; 4 pictures × 3 s), a dot was presented for 4 s followed by the rating scale for 2 s. Each block was followed by a crosshair, presented for 12–20 s, as a baseline. During presentation of the response cue, the participants were asked to judge whether or not the presented pictures were sleep-related. The participants were instructed to press the left button with their index finger for sleep-related pictures, and to press the right button with their middle finger if the pictures were not sleep-related.

### MRI data acquisition

A 3-Tesla whole-body Siemens scanner (TrioTim Syngo; Siemens, Erlangen, Germany) with a 12-channel birdcage head coil was used for functional image acquisition with an interleaved T2-weighted echo-planar imaging gradient echo sequence (repetition time = 3,000 ms, echo time = 30 ms, flip angle = 90°; slice thickness = 3.0 mm; in-plane resolution = 3.4 × 3.4 mm; field of view = 220 mm; matrix size = 64 × 64). The experiment was performed in an fMRI room on an 8-inch screen, and the stimuli were presented using DMDX software. For each participant, 174 functional volumes were acquired. Anatomical images were also acquired using a T1-weighted 3D gradient echo pulse sequence with magnetization-prepared rapid gradient echo (repetition time = 1,670 ms, echo time = 1.89 ms, flip angle = 9°, slice thickness = 1.0 mm, in-plane resolution = 1 × 1 mm, field of view = 250 mm, matrix size = 256 × 256). The method of MRI data acquisition process was cited from Kim et al. study^16^.

### fMRI preprocessing and analysis

The fMRI data were analyzed using SPM12 (Wellcome Department of Cognitive Neurology, London, UK). The first six volumes from all runs were discarded to prevent the non-equilibrium effect. The fMRI volumes were slice-time corrected. To correct for inter-scan rigid body motion, the volumes were realigned to the first image in the time series. After realignment, the volumes images were co-registered to the T1-weighted anatomical images and normalized to Montreal Neurological Institute space using a transformation matrix derived from T1 anatomical image segmentation. After normalization, the volumes were spatially smoothed using a 6-mm full width at half maximum isotropic Gaussian kernel, to compensate for residual inter-participant variability and increase statistical sensitivity. The resulting fMRI time series was high-pass filtered using a cut-off time of 128 s to remove low-frequency drifts. An artifact detection tool (http://www.nitrc.org/projects/artifact_detect/) was used to identify outlier volumes. Volumes with significant head motion (> 2 mm composite motion or large global mean intensity (i.e., a large difference in global mean intensities across functional volumes > 3 SD) were identified for each participant. The outlier volumes did not exceed 20% of the total volume in any participant, so no volumes were excluded from the final analysis.

A voxel-based general linear model was used to estimate parameters, including six motion parameters. Outliers were treated as covariates of no interest. Two regressors were used to model the sleep-related and neutral stimuli. The rating and fixation were also modeled to reduce the residuals. The design matrix was temporally convolved using a canonical hemodynamic response function to improve fit. Finally, contrast images associated with the conditions of interest [sleep-related or neutral stimuli] were created. The above method description of fMRI data preprocessing was cited from Kim et al. study^16^.

To evaluate differences in brain responses to sleep-related pictures between CID patients and GS, the contrast images [sleep-related pictures vs. neutral pictures] were analyzed using a whole-brain two-sample t-test. The significance level was set at *p* < 0.05, and was corrected for multiple comparisons using the false discovery rate (FDR). We extracted parameter estimates from brain regions showing differences in responses to sleep-related pictures (vs. neutral pictures) between the two groups.

### Statistical analysis

Continuous and categorical variables were analyzed using the *t* test and chi-square test, respectively, performed with SPSS Statistics software (version 24.0; IBM Corp., Armonk, NY, USA).

**Figure 3:**
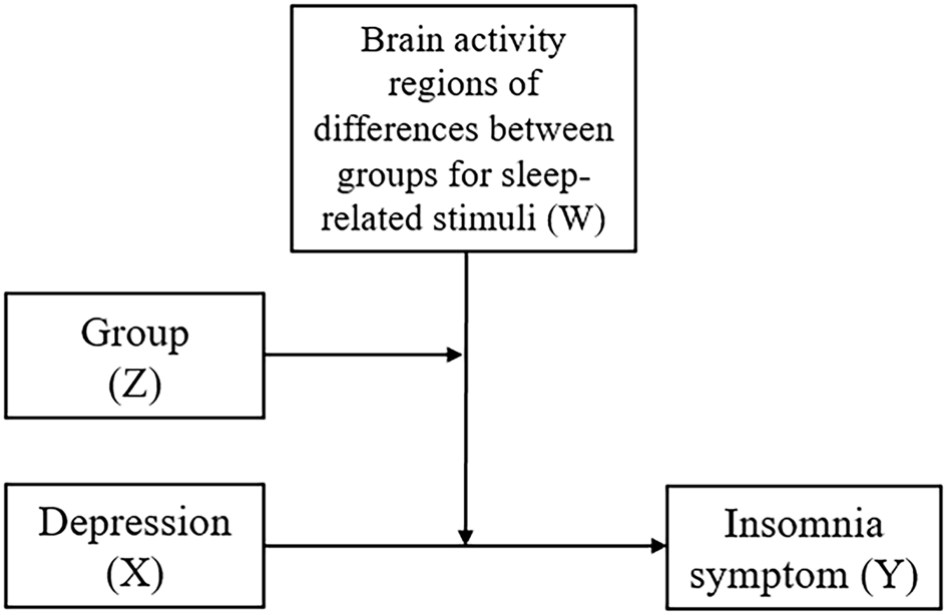
Moderated moderation model. A moderated moderation analysis was performed using the PROCESS macro (v3.0) for SPSS. In the model, group was entered as a moderator (Z) of the moderating effect of brain activity in response to sleep-related stimuli (W) on the association between sleep disturbances (dependent variable, Y) and depression (independent variable, X).

A moderated moderation analysis was performed to assess the moderating role of brain responses to sleep-related pictures in the association between depressive symptoms and sleep disturbance in CID patients and GS, using the PROCESS macro (v3.0) for SPSS^46^. The responses to sleep-related pictures and groups (CID vs. GS) were used as continuous and categorical moderators, respectively (Fig. 3). Depressive symptoms (BDI score) and sleep disturbance (sleep latency from the sleep diary) were included as independent and dependent variables, respectively. In between-group comparisons, behavioral and neuroimaging data were analyzed by adjusting for age, which could affect sleep disturbance. Conditional process analysis was performed using bootstrapping resampling (10,000 samples) to estimate 95% confidence intervals. Significant interactions were further tested using the Johnson–Neyman procedure implemented in PROCESS.

## Data Availability

All data generated or analysed during this study are included in this published article.
